# Axial Compression Behavior of Circular Seawater and Sea Sand Concrete Columns Reinforced with Hybrid GFRP–Stainless Steel Bars

**DOI:** 10.3390/ma17081767

**Published:** 2024-04-11

**Authors:** Hongwei Wang, Jinjin Xu, Jiuzhang Zhao, Xiaoyan Han, Kaiming Pan, Rena C. Yu, Zhimin Wu

**Affiliations:** 1 School of Civil Engineering and Architecture, Zhejiang University of Science and Technology, Hangzhou 310023, China; 2 School of Civil Engineering, Tianjin Ren’ai College, Tianjin 301636, China; 3 State Key Laboratory of Coastal and Offshore Engineering, Dalian University of Technology, Dalian 116024, China; 4 ETSI de Caminos, Canales y Puertos, University of Castilla-La Mancha, 13001 Ciudad Real, Spain

**Keywords:** hybrid reinforcement, column, axial compression, ductility

## Abstract

The ductility of FRP-reinforced concrete structures is reduced by the brittleness of FRP bars. To address this issue, this study employs the hybrid reinforcement of stainless steel (SS) and GFRP bars to enhance the ductility of concrete columns. A total of 21 axially compressed seawater and sea sand concrete (SWSSC) circular columns are fabricated, including 15 hybrid GFRP and SS bar-reinforced SWSSC (GFRP-SS-SWSSC) columns, 3 GFRP bar-reinforced SWSSC (GFRP-SWSSC) columns, and 3 SS bar-reinforced SWSSC (SS-SWSSC) columns. The test results are analyzed in terms of failure mode, load–axial displacement curve, bearing capacity, and ductility. Results show that GFRP-SWSSC columns suffer brittle failure, while GFRP-SS-SWSSC columns and SS-SWSSC columns demonstrate ductile failure characteristics. Furthermore, the hybrid reinforcement contributes to an improvement in the bearing capacity of the columns. A calculation equation for the bearing capacity of axially compressed columns was established, providing reasonable predictions of bearing capacities, with a design compressive strain of 2000 με for GFRP bars. It was found that hybrid reinforcement enhanced the ductility of GFRP-SWSSC columns. In addition, when the percentage of the SS reinforcement ratio reaches 50%, the ductility indexes of the GFRP-SS-SWSSC columns closely approach those of the SS-SWSSC columns.

## 1. Introduction

For structures located in harsh environments such as coastal and island regions, using FRP reinforcement as a substitute for steel reinforcement can effectively address durability issues caused by steel corrosion. Currently, research on FRP-reinforced concrete (FRP-RC) beams is quite extensive. However, research on the axial compression behavior of FRP-RC columns is limited due to the lack of standardized testing methods for the compressive performance of FRP bars and the relatively lower compressive strength of FRP bars. At present, studies concerning axially compressed FRP-RC columns mainly focus on the load-bearing capacity and the contribution of FRP bars to the bearing capacity.

De Luca et al. [1] carried out axially compressed tests for GFRP-reinforced concrete (GFRP-RC) columns. Their results indicated that GFRP bars contributed below 5% of the bearing capacity, obviously lower than the 12% contribution of steel bars at the identical reinforcement ratio. Furthermore, decreasing the stirrup spacing from 305 mm to 76 mm had a negligible impact on the bearing capacity but significantly improved the ductility of the columns. Hadhood et al. [2] found that the influence of reinforcement ratio on the capacity of FRP bars reinforced with high-strength concrete columns is not significant, and FRP bars contributed approximately 5% of the bearing. Results in Tobbi et al. [3] indicated that for well-confined GFRP-RC axially loaded columns, GFRP bars could contribute 10% of the bearing capacity. In addition, the bearing capacity can be reasonably calculated when the GFRP bars’ compressive strength is taken as 35% tensile strength. Afifi et al. [4] analyzed the axially compressed performance of circular GFRP-RC columns. The results showed that the contribution of GFRP bars to the bearing capacity was between 5% and 10%. Furthermore, this study confirmed that the compressive design strength for GFRP bars at 35% of its tensile strength recommended by Tobbi et al. [3] could accurately predict the bearing capacity. Afifi et al. [5] also investigated the axial compression behavior of circular concrete columns reinforced with sand-coated CFRP bars. Results indicated that CFRP bars could contribute 12% of the bearing capacity. Moreover, the peak load was reasonably calculated by adopting the CFRP bar’s compressive strength at 25% of its tensile strength. Research by Hadi and Youssef [6] indicated that the bearing capacity of the GFRP-RC column was 4.8% smaller than that of the steel-reinforced concrete column. Elmessalami et al. [7] found that the axial bearing capacities of GFRP-RC columns and BFRP-RC columns were almost identical, with longitudinal bars contributing to approximately 11% of the bearing capacity. Hadi et al. [8] investigated the axially compressed behavior of GFRP-reinforced high-strength concrete columns. It was noted that directly substituting FRP bars for steel bars of the same reinforcement area would result in a 50% reduction in the bearing capacity. Hasan et al. [9] proposed an equation to compute the axial bearing capacity of FRP-RC columns. It was found that the bearing capacity can be reasonably predicted by calculating the contribution of FRP bars using the strain and elastic modulus.

In addition to studying the contribution of FRP bars to the peak load, the FRP bars’ design strains in axially compressed concrete columns have also been analyzed. Tobbi et al. [10] conducted axial compression tests on confined GFRP-RC and CFRP-RC columns. The results showed that accurate predictions of the bearing capacity can be achieved by taking the design strain of FRP bars at 0.003, which is the concrete compressive strain corresponding to the peak load due to the strains of FRP bars and concrete being approximately equal in axially compressed columns. Youssef and Hadi [11] and Hadhood et al. [12] made a study of the axially compressed behavior of GFRP-RC columns. They discovered that the design strain of 0.003 for FRP bars suggested by Tobbi et al. [10] can reasonably predict the bearing capacity. Mohamed et al. [13] and Xue et al. [14] found that GFRP bars’ design strain of 0.002 could accurately predict the bearing capacity for axially compressed GFRP-RC columns. Hadhood et al. [15,16] investigated the axially compressed behavior of CFRP bars reinforced with ordinary and high-strength concrete columns. Results indicated that the bearing capacity could be reasonably predicted by setting the design strain at 0.0035 for CFRP bars, which assumes that the design strain of CFRP bars is equal to the ultimate compressive strain of concrete. However, the ultimate compressive strain is less than 0.0035 for high-strength concrete. Xiong et al. [17] examined the axial compression performance of SWSSC columns reinforced with BFRP bars. Results showed that for confined concrete columns, adopting a design strain of 0.003 for BFRP bars provided a more accurate prediction of the bearing capacity.

The hybrid reinforcement of FRP and steel bars has been proposed to improve the ductility of the members due to the brittleness of FRP bars and the quasi-brittleness of concrete. It was noted that the hybrid reinforcement can greatly enhance the ductility of FRP-RC beams [18,19,20]. Nevertheless, few studies on the behavior of axially compressed concrete columns reinforced with hybrid reinforcement have been reported. Wright and Pantelides [21] studied the axially compressed behavior of concrete columns longitudinally and transversely reinforced with hybrid GFRP and stainless steel (SS) bars. They found that the ductility of hybrid-reinforced concrete columns was superior than that of GFRP-RC columns. Tahir et al. [22] developed a confinement model through the analysis of results from steel bars and FRP stirrups in reinforced concrete columns. This model can reasonably calculate the stress–strain curve and bearing capacity. Sun et al. [23,24] investigated the compression behavior of concrete columns with reinforcement configurations including steel, hybrid, and steel–FRP composite bars. Their results indicated that the residual deformations of the hybrid-reinforced and SFCB-reinforced concrete columns were smaller than those of steel-reinforced columns, and the post-yielding stiffness of the columns reinforced with hybrid steel and FRP bars increased by 27%.

The good corrosion resistance of the FRP bar is applicable for seawater and sea sand concrete (SWSSC) members. Current research has found that chloride ions in seawater and sea sand have little effect on FRP reinforcement. Moreover, for coastal and island buildings, the use of SWSSC not only reduces the transportation cost and shortens the construction period, but also saves freshwater and river sand resources. However, the hybrid reinforcement of FRP and ordinary steel bars cannot be employed in SWSSC structures because of the easy corrosion of steel bars. SS bars possess excellent corrosion resistance and favorable ductility but are more expensive. Therefore, for SWSSC members, hybrid FRP and SS bars can be used to improve ductility. Xu et al. [25] investigated the axial compression performance of SWSSC square columns reinforced with hybrid FRP and SS bars. It was found that ductility can be enhanced by hybrid reinforcements. In addition, based on the statistically measured compressive strains at the peak load of GFRP bars, it is recommended that the design strain of GFRP bars be taken as 0.002.

Different from Xu et al. [25], this paper investigates the axial compression performance of hybrid FRP and SS bar-reinforced SWSSC columns with a circular cross-section. In addition, to fully utilize the yield stage of SS bars, a lower yield strength of SS bars is adopted compared with that of Xu et al. [25]. The axial compressive performance of GFRP-reinforced and SS-reinforced columns is comparatively analyzed. The load–axial displacement curve, failure mode, bearing capacity, and ductility of SWSSC columns are discussed. In addition, an equation for calculating the bearing capacity is proposed and compared with the results of Xu et al. [25].

## 2. Test Procedure

### 2.1. Materials

#### 2.1.1. Rebars

**Figure 1:**
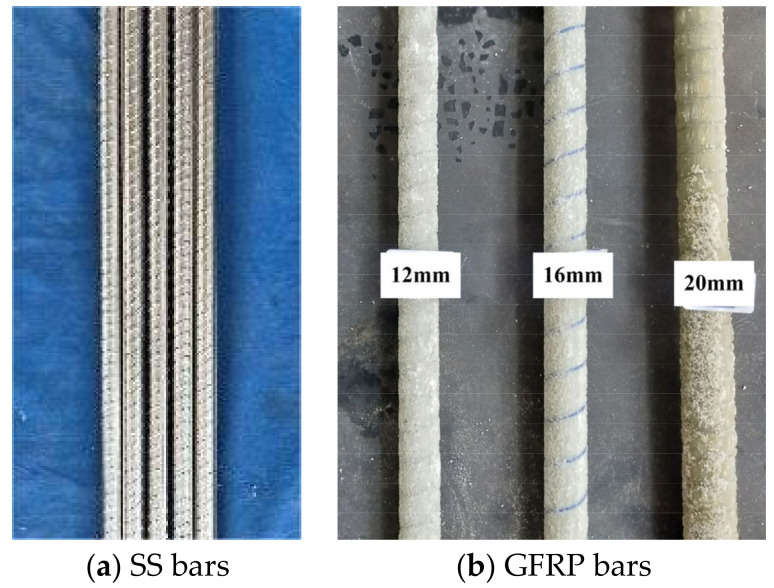
Reinforcing bars.

The ribbed 304 SS bars from Taizhou Kaiding Stainless Steel Products Co., Ltd., Taizhou, China, with a diameter of 16 mm and the sand-coated GFRP bars from Shenzhen Haichuan New Materials Technology Co., Ltd., Shenzhen, China, with diameters of 12 mm, 16 mm, and 20 mm were adopted, as illustrated in Figure 1.

**Figure 2:**
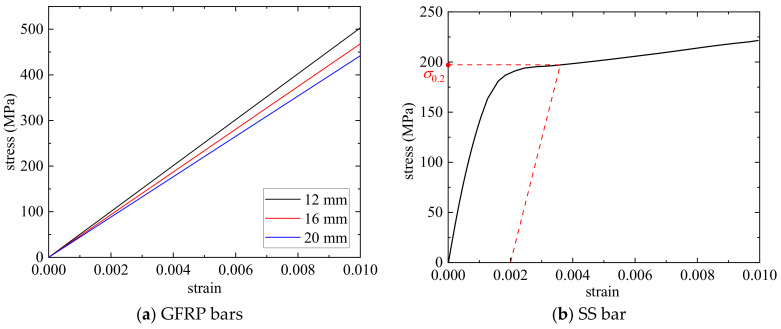
The stress–strain curves of reinforcing bars.

The mechanical properties of GFRP and SS bars were tested based on ASTM D 7205 [26] and GB 228.1 [27], respectively. It should be noted that the strains of reinforcing bars can only be measured to 0.01 due to the small range of the extensometer, but the tensile strength can be collected after removing the extensometer. As shown in Figure 2a, the stress–strain curves of all the GFRP bars show linear relationships. As shown in Figure 2b, the conditional yield strength *σ*_0.2_ was used considering there was no yield stage for the SS bar. The *σ*_0.2_ of SS bars is 194 MPa, and the elastic modulus is 172 GPa. The utilization of SS bars with lower yield strength is aimed at fully using the plasticity of SS bars to enhance the ductility of columns. The mean values, standard deviation, and coefficient of variation (COV) of tensile strength (*f*_fu_) and elastic modulus (*E*_f_) of GFRP bars are presented in Table 1. From Table 1, it can be observed that, due to size effects, there are slight differences in the *f*_fu_ and *E*_f_ of GFRP bars with different diameters.

#### 2.1.2. SWSSC

The natural seawater from Dalian City and the sea sand from Qingdao City, China, were adopted in this test. The primary compositions of the seawater are presented in Table 2. The sea sand’s fineness modulus is 2.61, which was measured based on GB/T 14684-2011 [28], and the sand is medium sand.

Crushed limestone (5–20 mm) was adopted as coarse aggregate, and Portland 42.5 cement was also adopted. Three concrete grades, C20, C30, and C40, were designed according to GB 50010-2010 [29], and the mixture proportions are presented in Table 3. These three concrete strength grades designed in this paper allow for the development of an equation for calculating the bearing capacity applicable to ordinary-strength concrete and can be applied to island and reef areas where high-rise structures are generally not constructed. After 30-day curing, the mechanical properties of the SWSSC were measured according to GB/T 50081-2019 [30], and the mean values, standard deviation, and COV of cubic compressive strength *f*_cu_, prism compressive strength *f*_c_, elastic modulus *E*_c_, and Poisson’s ratio *ν* are shown in Table 4.

### 2.2. Specimen Design

**Figure 3:**
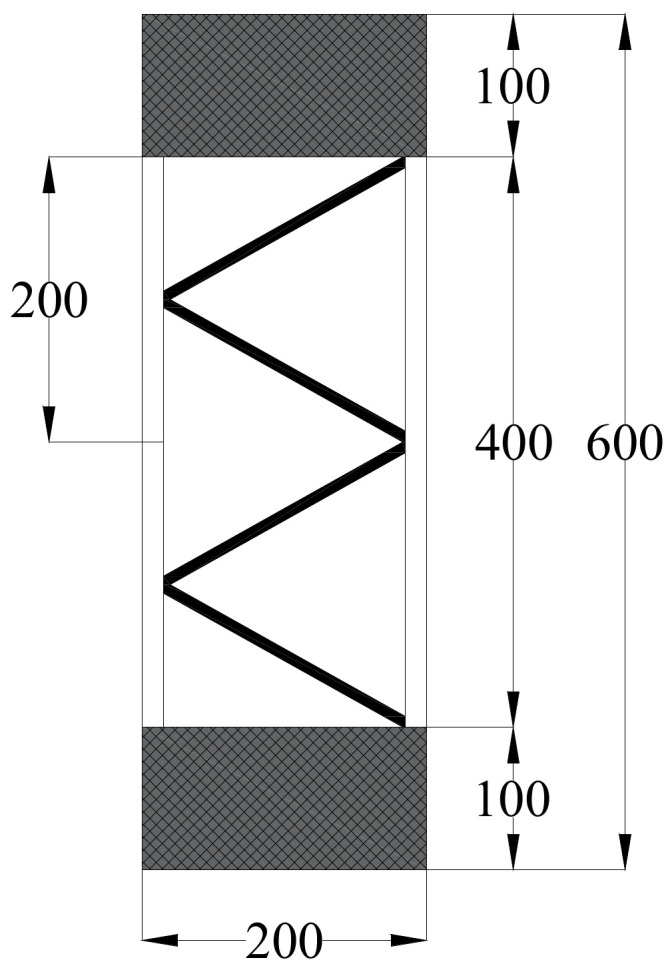
Column sizes (mm).

A total of 21 SWSSC circular columns are fabricated, including 3 GFRP bar-reinforced SWSSC (GFRP-SWSSC) columns, 3 SS bar-reinforced SWSSC (SS-SWSSC) columns, and 15 hybrid GFRP-SS-reinforced SWSSC (GFRP-SS-SWSSC) columns. The height and diameter of the columns are 600 mm and 200 mm, respectively, and the concrete cover is 15 mm, considering the good corrosion resistance of GFRP and SS bars. As shown in Figure 3, the upper and lower 100 mm of the column are wrapped externally with two layers of CFRP sheets for loading. The GFRP spiral stirrups are arranged in the middle 400 mm of the column, with a diameter of 6 mm and a spacing of 200 mm. The reason for the large stirrup spacing is that the stirrups are construction reinforcements, and the confining effect on concrete columns is not considered.

**Figure 4:**
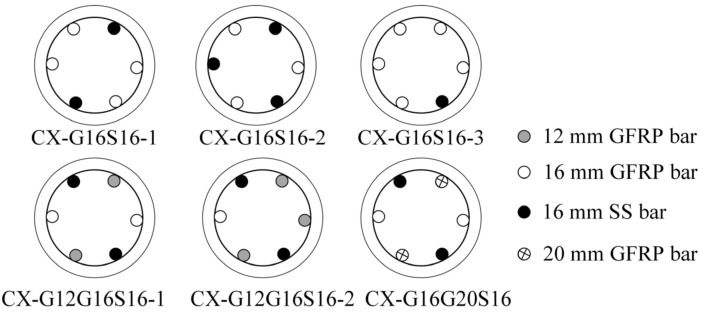
The section reinforcements for hybrid-reinforced SWSSC columns.

Six reinforcing bars are symmetrically arranged in each column. Four reinforcement ratios are designed, which are 3.00%, 3.28%, 3.84%, and 4.56%. For hybrid-reinforced SWSSC columns, four ratios (*A*_SS_/*A*_GFRP_) between the reinforcement areas of SS and GFRP bars are designed to analyze the effect of replacing GFRP bars with SS bars on the ductility improvement of GFRP-SWSSC columns. The reinforcement details for hybrid-reinforced SWSSC columns are shown in Figure 4. The column identifier is written “concrete strength + bars’ type + bars’ diameter”. For example, the C30G16G20S16 represents a C30 SWSSC column, and the reinforcements are 2 GFRP bars with a diameter of 16 mm, 2 GFRP bars with a diameter of 20 mm, and 2 SS bars with a diameter of 16 mm. The reinforcement details of all the columns are shown in Table 5.

### 2.3. Instrumentation

**Figure 5:**
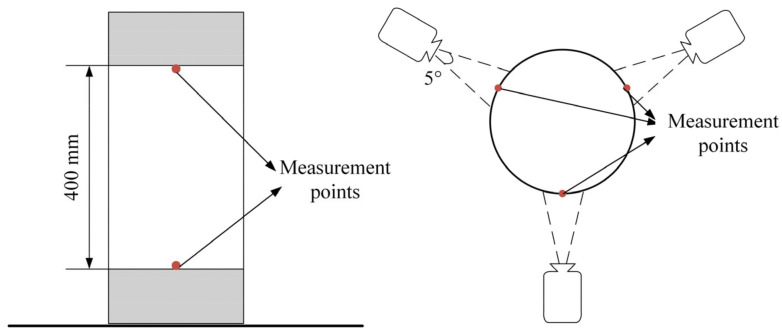
The DIC observation diagram.

Two longitudinal bars of each diameter were selected, and a resistance strain gauge was pasted on the mid-height of these bars to measure the strain of the longitudinal bars. The concrete strain and the axial displacement for the middle 400 mm of the column were measured by digital image correlation (DIC). To achieve the calibration of the pixel-to-metric scale, a calibration object with known dimensions should be placed in the speckle region of the specimen. In this study, the unconfined segment of the column measuring 400 mm in length is used as the calibration distance standard. Speckles were sprayed on the surface, and the measurement range for axial displacement is 400 mm high in the middle of the column. The measurement points are shown in Figure 5. As a non-contact measurement method, DIC eliminates the need for installing displacement meters or pasting strain gauges to the column surface. In addition, the measurement accuracy of DIC is higher due to its pixel-level image calculation. The DIC observation diagram is shown in Figure 5.

**Figure 6:**
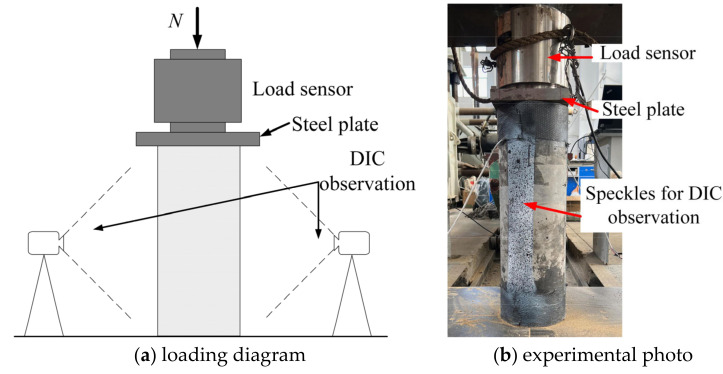
The test setup.

Prior to testing, we centered and leveled the column to prevent eccentric loading. Tests were carried out using a 5000 kN hydraulic machine from Structural Hall of Dalian University of Technology in China, employing a controlled displacement rate of 0.2 mm/min. A spherical joint is equipped on the upper part of the testing machine to prevent eccentric loading. The 3000 kN load sensor was adopted to determine the axial load. The measurement instruments were linked to a dynamic data acquisition device for continuous real-time data collection, and the frequency was 50 Hz. The loading diagram and the photo of the test setup are illustrated in Figure 6.

## 3. Test Results and Discussions

### 3.1. Load–Axial Displacement Curve and Failure Mode

**Figure 7:**
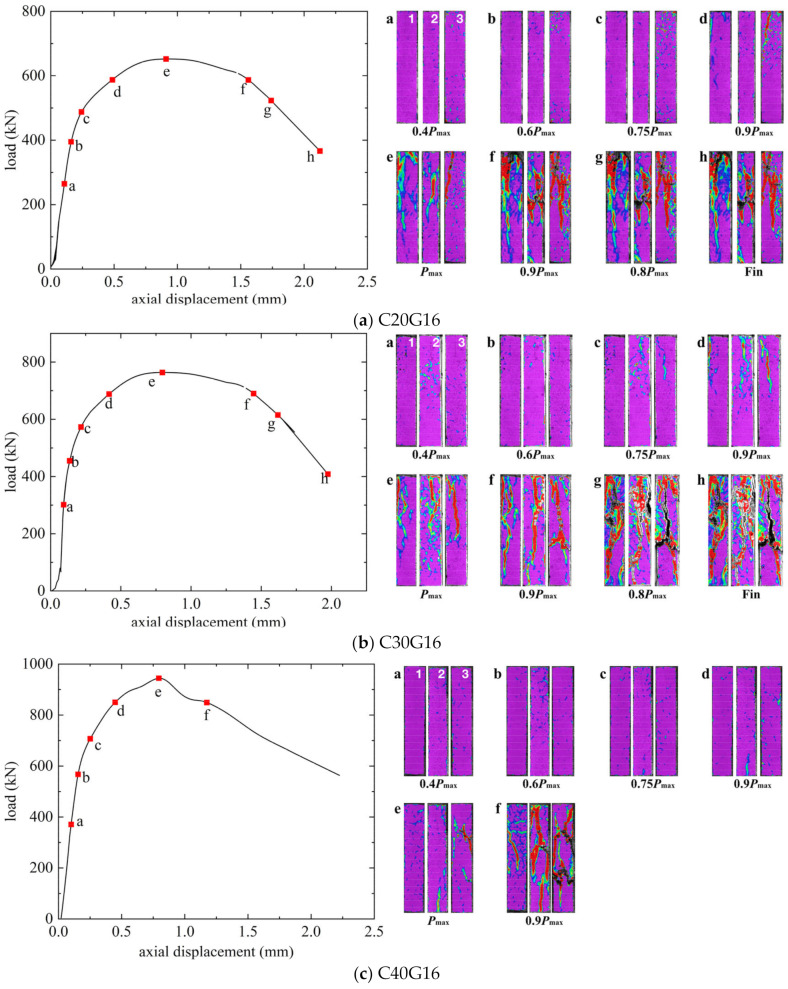
Load–axial displacement curves for GFRP-SWSSC columns.

**Figure 8:**
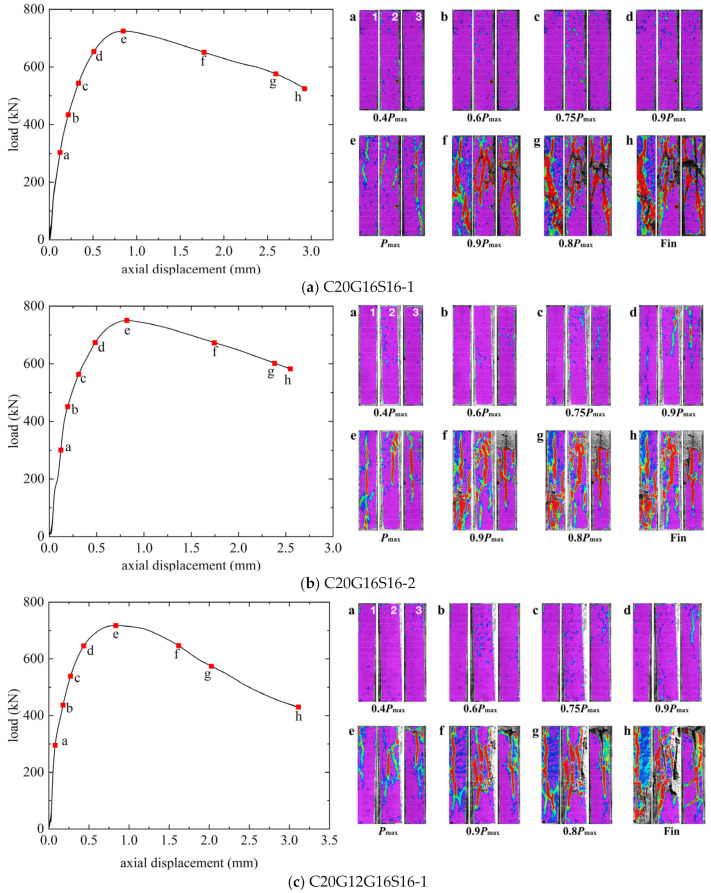

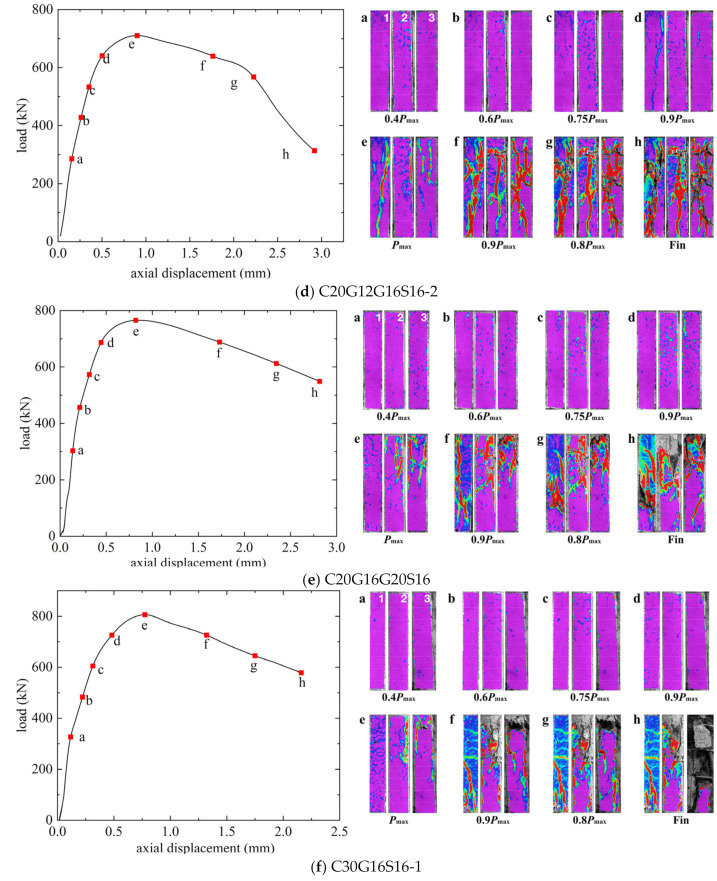

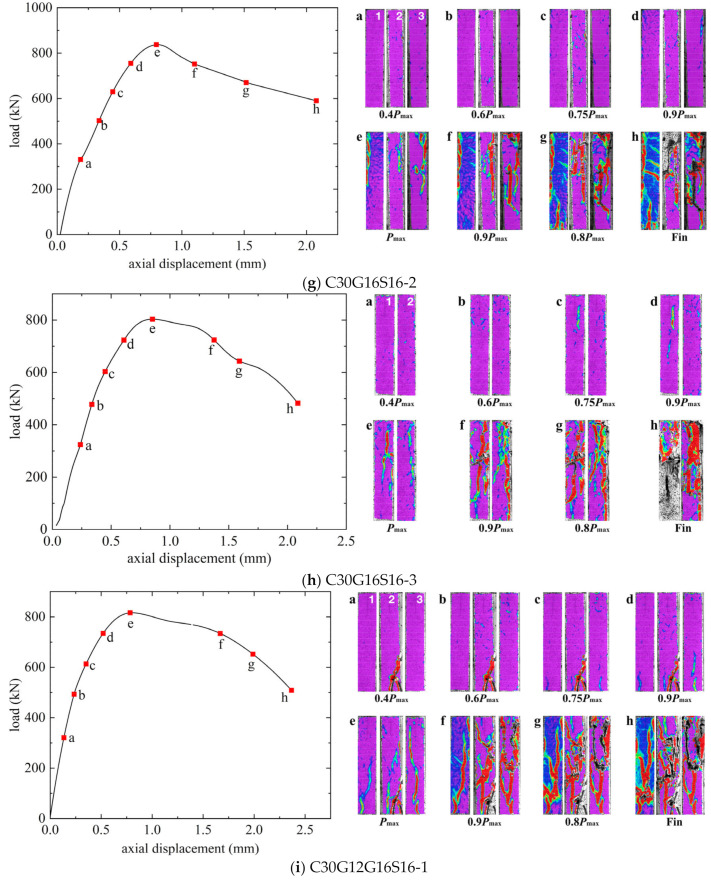

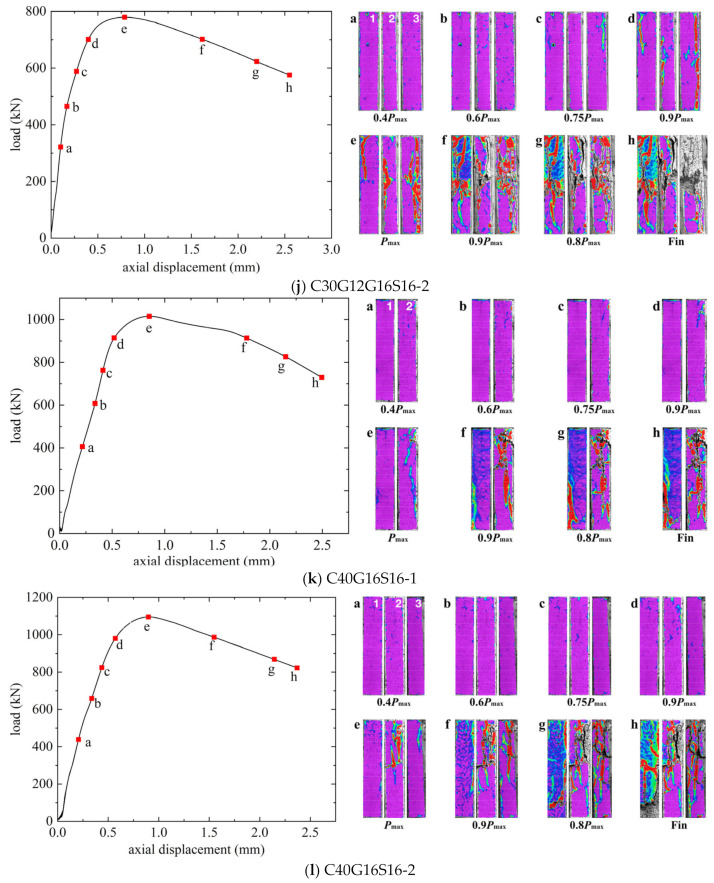

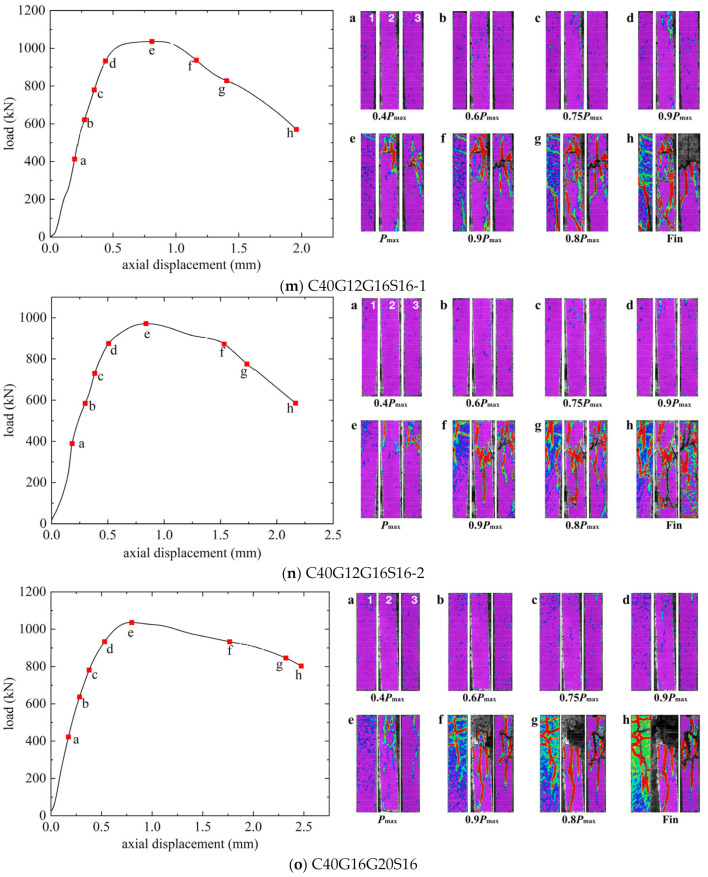
Load–axial displacement curves for GFRP-SS-SWSSC columns.

**Figure 9:**
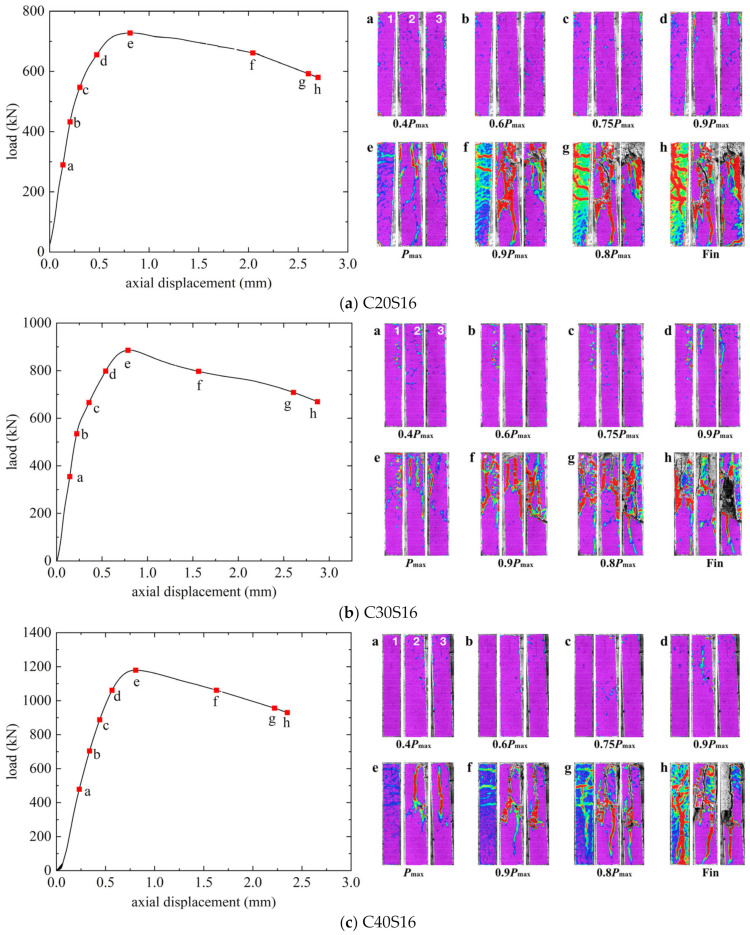
Load–axial displacement curves for SS-SWSSC columns.

The load–axial displacement curves and the crack propagations observed with DIC of GFRP-SWSSC columns, GFRP-SS-SWSSC columns, and SS-SWSSC columns are presented in Figure 7, Figure 8, and Figure 9, respectively. The numbers 1–3 in the figures refers to the three surfaces of the column captured by the three cameras in DIC. In the first period of loading, the load–axial displacement curves exhibited similar behavior, characterized by a generally linear increase in axial displacement with the applied load. And no cracks can be found. Vertical cracks were observed on the surface of the column when the load reached 0.75*P*_max_. Subsequently, the axial displacement increased rapidly while the load increased slowly until the peak load was reached. The axial displacements (Δ*u*) corresponding to the peak load for all the columns are presented in Table 6. Note that the Δ*u* for all columns remains relatively close, ranging from 0.78 to 0.95 mm.

After reaching the peak load, the behavior of the descending part of the load–axial displacement curve varied among SWSSC columns reinforced with different reinforcement types. Note from Figure 7 that the load on the GFRP-SWSSC column gradually decreased to approximately 0.9*P*_max_ before quickly dropping to about 50% of the peak load within a few seconds, indicating a brittle failure characteristic. In contrast, as depicted in Figure 8 and Figure 9, the curves for GFRP-SS-SWSSC columns and SS-SWSSC columns show a gradual decrease in load during the descending part, which differs from that of GFRP-SWSSC columns.

Based on the images captured through DIC, it can be seen that limited vertical cracks initiated when the load increased to 0.75*P*_max_. Moreover, the vertical cracks increased, and the cracks developed and became evident when the load reached 0.9*P*_max_. In addition, for the GFRP-SS-SWSSC and SS-SWSSC columns, the SS bars yielded at approximately this load level. After the SS bars yielded, the load did not decrease but increased slowly, indicating that GFRP bars exhibit good resistance to the axial load. Subsequently, the cracks gradually widened and developed when the load reached *P*_max_. Because of the relatively low slenderness ratio, longitudinal bars did not buckle at the peak load, and the failure mode was concrete crushing. It should be noted that during the observation of the DIC for C30G16S16-3, only two image directions were available due to lighting conditions. From Figure 7, Figure 8 and Figure 9, it can be observed that the distribution of cracks on different surfaces of the column is significantly different at failure. The reason for this is that the heterogeneity of concrete leads to inevitable eccentricity during compression loading.

**Figure 10:**
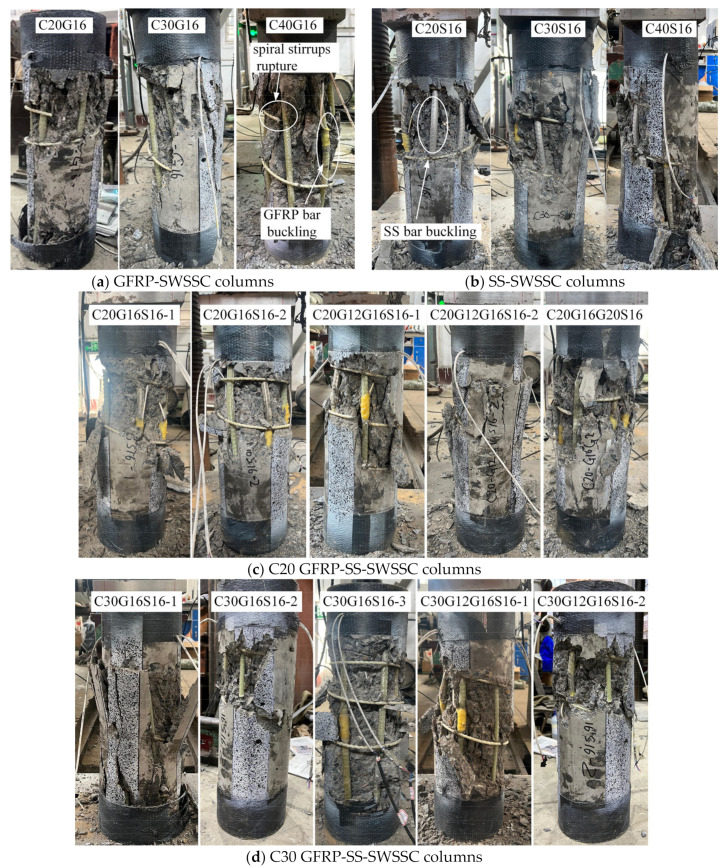

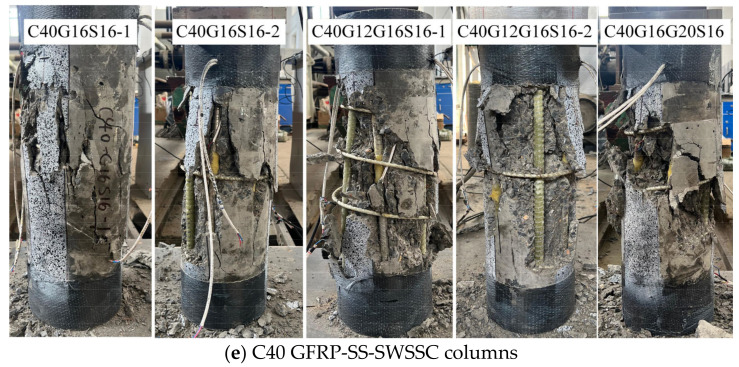
Failure modes for all the columns.

After reaching the peak load, the concrete cover spalled off. And some GFRP and SS longitudinal bars buckled due to the absence of the confinement provided by the concrete cover. In addition, some GFRP stirrups experienced fractures. The ultimate failure modes of all columns are illustrated in Figure 10.

### 3.2. Bearing Capacities

#### 3.2.1. Experimental Bearing Capacities

**Figure 11:**
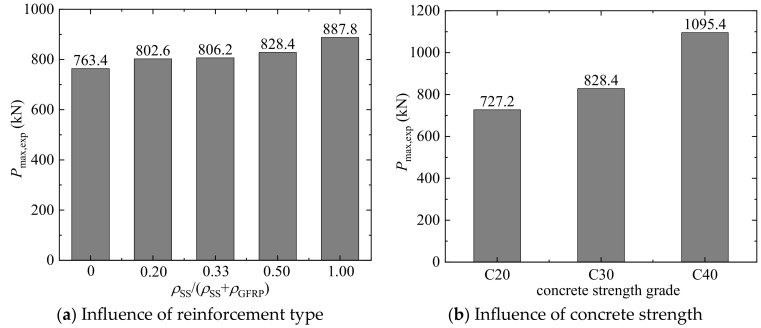
Bearing capacities of columns.

The experimental bearing capacities (*P*_max,exp_) are provided in Table 6. Figure 11a illustrates the influence of reinforcement type on the bearing capacity under the concrete strength grade of C30 and the reinforcement ratio of 3.84%. In this figure, the value of *ρ*_SS_/(*ρ*_SS_ + *ρ*_GFRP_) equal to 0.0 represents the GFRP-SWSSC column, while the value of *ρ*_SS_/(*ρ*_SS_ + *ρ*_GFRP_) equal to 1.0 represents the SS-SWSSC column. It is observed that increasing the reinforcement ratio of SS bars leads to an increase in the bearing capacity. When the percentage of reinforcement ratio of SS bars increased from 0 to 0.20, 0.33, 0.50, and 1.00, the axial compressive bearing capacity increased by 5%, 6%, 9%, and 16%, respectively. Furthermore, it can be seen from Table 6 that for the same reinforcement ratio and concrete strength, SS-SWSSC columns have the highest bearing capacity, followed by the GFRP-SS-SWSSC columns, and then the GFRP-SWSSC columns. This indicates that substituting SS bars for part GFRP bars increases the contribution of the longitudinal bars to *P*_max,exp_ due to the higher elastic modulus of SS bars.

Figure 11b presents the bearing capacity of G16S16-2 columns under three concrete strength grades. It is observed that as the concrete strength increased from 20.74 MPa to 27.36 MPa and 36.90 MPa, the bearing capacity increased by 14% and 51%, respectively. This shows the significant influence of concrete strength on the bearing capacity of axially compressed columns. It is worth noting that this impact is similarly evident in the other columns, as indicated in Table 6. In addition, compared to the reinforcement type, the influence of concrete strength on bearing capacity is more notable. This is because the bearing capacity of axial compression columns is primarily provided by concrete, given the relatively small area of longitudinal reinforcement. The reinforcements are mainly to improve the ductility.

#### 3.2.2. Contribution of Longitudinal Bars to Bearing Capacity

**Figure 12:**
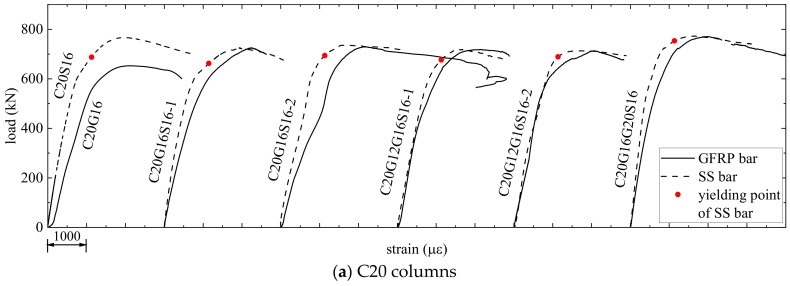

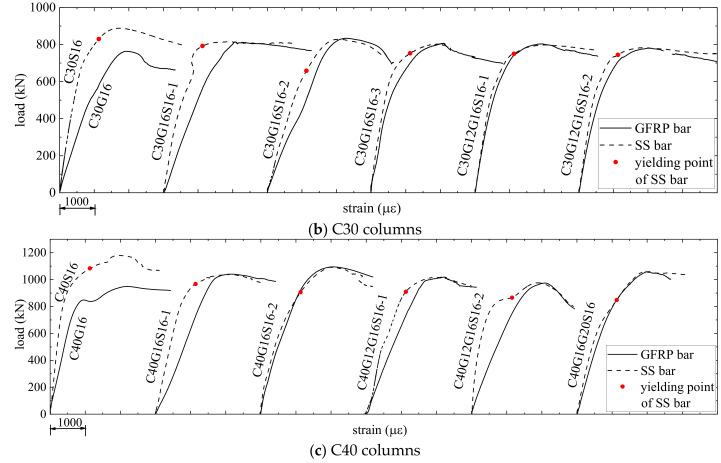
The strains of longitudinal bars.

The strains of longitudinal bars for all the columns are shown in Figure 12. It can be observed that the strains of GFRP bars and SS bars follow a similar trend with changes in load. The strain of GFRP bars (*ε*_fp_), strain of SS bars (*ε*_sp_), and concrete strain (*ε*_cp_) at peak are summarized in Table 6. From Table 6, it can be seen that the compressive strains of GFRP bars, SS bars, and concrete at peak are basically equal, which indicates that the GFRP and SS bars are perfectly bonded to the concrete. The yielding points of SS bars are marked on the load-strain curves for SS bars in Figure 12, with the yield strain for the SS bar being 1128 με. Note that the load increased slightly after the SS bars yielded. With the same load, the strains of SS bars are smaller than those of GFRP bars, suggesting that the SS bars provide greater axial stiffness. Moreover, under the same strains of longitudinal bars, the stresses of SS bars are higher than those of GFRP bars, indicating SS bars contributed more than GFRP bars to the peak load. In addition, the strains of concrete and bars at peak are around 2000 με, which is similar to current studies [13,14,25].

The bearing capacities provided by the longitudinal bars (*P*_bar_) and their contribution to the bearing capacity (*P*_bar_/*P*_max,exp_) are presented in Table 6. It can be found that the higher elastic modulus of SS bars leads to a greater contribution to *P*_max,exp_ compared to GFRP bars. The average values of *P*_bar_/*P*_max,exp_ for GFRP-SWSSC columns, GFRP-SS-SWSSC columns, and SS-SWSSC columns are 15%, 19%, and 25%, respectively. This also indicates the fact that hybrid reinforcement can enhance the contribution of longitudinal bars.

#### 3.2.3. Prediction of Bearing Capacity

The current studies [3,4,10,25] indicate that neglecting the contribution of FRP bars in the calculation of the bearing capacity for axially compressed FRP-RC columns results in conservative results, while considering the contribution of FRP bars provides accurate predictions [3,4,10,25]. Therefore, in this study, the theoretical calculation of the *P*_max_ takes into account the contribution of GFRP bars.

Since the strain of the GFRP bar at the peak load is approximately 2000 με, it is reasonable to consider the design strain for GFRP longitudinal bars as 2000 με. And the SS bar will already yield when the peak load is reached. Therefore, when calculating the bearing capacity, the yield strength of SS bars is employed. Based on the force balance and assuming that GFRP bars behave as an ideal linear elastic material under compression, the bearing capacity calculation equation for GFRP-SS-SWSSC columns can be derived as follows:

(1) $$P_{\max,\mathrm{pre}}=\alpha_{1}f_{c}\left(A_{g}-A_{\mathrm{GFRP}}-A_{\mathrm{SS}}\right)+f_{y}A_{\mathrm{SS}}+0.002E_{\mathrm{GFRP}}A_{\mathrm{GFRP}}$$

where *α*_1_ is the reduction factor for concrete strength, taken as 0.85; *f*_c_ is the axial compression strength of SWSSC; *A*_GFRP_ and *A*_SS_ are the reinforcement areas of GFRP and SS bars, respectively; *f*_y_ is the SS bar’s yield strength; *E*_GFRP_ is the elastic modulus of the GFRP bar; and *A*_g_ is the area of the gross section. Additionally, Equation (1) can be used to calculate the axial compressive bearing capacities of the GFRP-SWSSC and SS-SWSSC columns.

**Figure 13:**
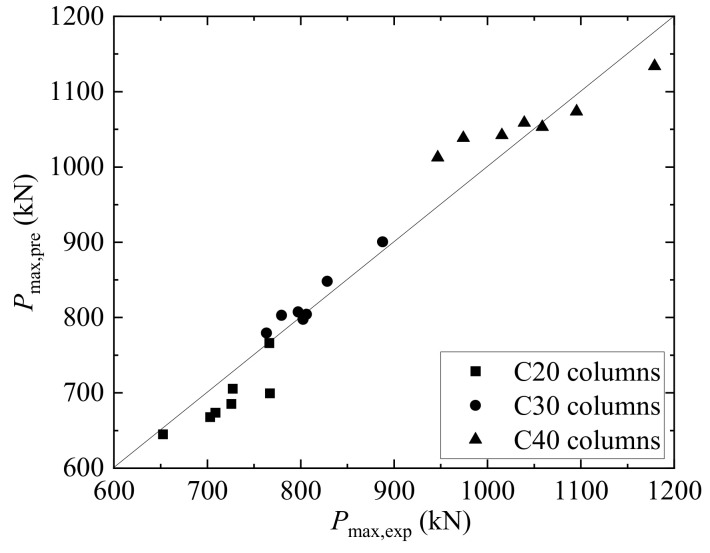
The comparison between the predicted and experimental bearing capacities.

The predicted bearing capacities (*P*_max,pre_) calculated using Equation (1) are presented in Table 6. It can be observed that the average ratio of predicted to experimental bearing capacities is 0.99. Furthermore, the predicted and experimental values are compared in Figure 13. The scatter of data points near the 45-degree line indicates that the proposed bearing capacity calculation equation accurately predicts the bearing capacity. It is noteworthy that the proposed bearing capacity equation is consistent with that presented by Xu et al. [25], indicating its applicability to both circular and rectangular concrete columns.

### 3.3. Ductility

**Figure 14:**
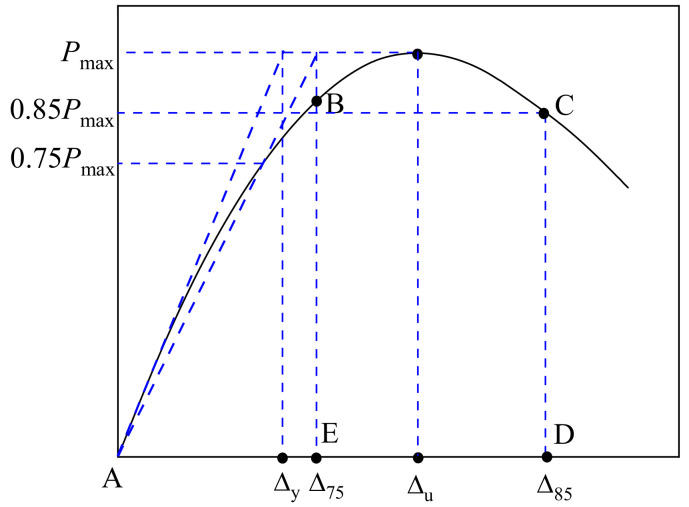
The determination of the ductility index.

The brittleness of FRP bars reduces the ductility of FRP-RC members. Therefore, this study employs a hybrid reinforcement approach with GFRP and SS bars to enhance the ductility of GFRP-RC axial compression columns. The energy ductility index *μ*_1_ [31,32,33,34] is adopted to analyze the ductility of axial compression columns, which is calculated as follows:

(2) $$\mu_{1}=\frac{E_{u}}{E_{y}}=\frac{S_{\mathrm{ABCD}}}{S_{\mathrm{ABE}}}$$

where, as illustrated in Figure 14, *S*_ABCD_ represents the area under the curve up to the point where the load reaches 0.85*P*_max_ within the post-peak descent part; *S*_ABE_ is the area under the curve when the displacement reaches Δ_75_; and Δ_75_ represents the displacement at the intersection of the line between the origin and 0.75*P*_max_ and the horizontal line at *P*_max_; and *E*_u_ and *E*_y_ are the energy corresponding to the ultimate state and the yield state, respectively.

In addition, the displacement ductility index *μ*_2_ is also used to analyze the ductility of the SWSSC columns, and *μ*_2_ is calculated by:

(3) $$\mu_{2}=\frac{\Delta_{85}}{\Delta_{y}}$$

where, as depicted in Figure 14, A is the origin; B and C are the intersection points of the Δ_75_ and Δ_85_ vertical lines with the curve, respectively; D and E are the points with displacement of Δ_75_ and Δ_85_ on the x-axis, respectively; Δ_y_ is the axial displacement corresponding to the limit of elastic behavior on the ascending part [35]; Δ_85_ is the axial displacement corresponding to the load reaching 0.85*P*_max_ within the post-peak descent part. The calculation parameters and ductility indexes for all the SWSSC columns are given in Table 7.

**Figure 15:**
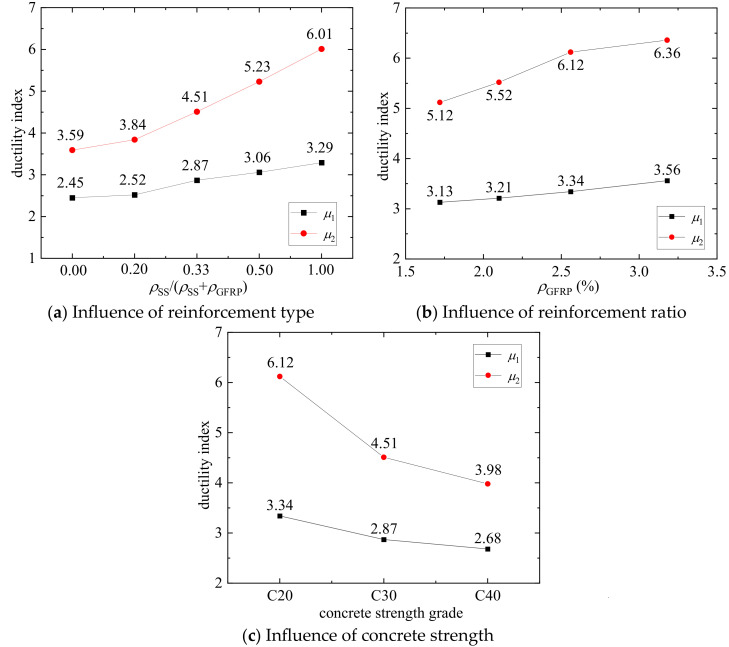
The ductility indexes of SWSSC columns.

Figure 15a shows the influence of reinforcement type on ductility for the C30 concrete strength grade and the reinforcement ratio of 3.84%. In the figure, the value of *ρ*_SS_/(*ρ*_SS_ + *ρ*_GFRP_) equal to zero represents the GFRP-SWSSC column, while the value of *ρ*_SS_/(*ρ*_SS_ + *ρ*_GFRP_) equal to one represents the SS-SWSSC column. Note that SS-SWSSC columns exhibit the highest ductility, followed by hybrid-reinforced SWSSC columns, while GFRP-SWSSC columns show the lowest ductility. The energy and displacement ductility indexes show the same trend. Increasing the *ρ*_SS_/(*ρ*_SS_ + *ρ*_GFRP_) from 0 to 0.2, 0.33, and 0.5 resulted in *μ*_1_ improvements of 3%, 17%, and 25%, respectively, and resulted in *μ*_2_ improvements of 7%, 25%, and 45%, respectively. This indicates that hybrid reinforcement can enhance the ductility of GFRP-SWSSC columns. From Table 7, it can be found that the GFRP-SS-SWSSC columns can achieve close ductility indexes to those of the SS-SWSSC columns when the percentage of reinforcement ratio of the SS bars is 0.5.

Figure 15b illustrates the effect of increasing the GFRP reinforcement ratio on the ductility of axially compressed columns with the concrete strength at C20 and the SS reinforcement ratio of 1.28%. Note that under the same reinforcement ratio of SS bars, increasing the reinforcement ratio of GFRP bars also improves the ductility of the columns. When the GFRP reinforcement ratio increases from 1.72% to 2.00%, 2.56%, and 3.28%, the *μ*_1_ improves by 3%, 7%, and 14%, respectively, and the *μ*_2_ improves by 8%, 19%, and 24%, respectively.

Figure 15c presents the variation in ductility indexes of G16S16-1 columns under different concrete strengths. With the same reinforcement ratio, the energy and displacement ductility indexes decrease with an increase in concrete strength. The *μ*_1_ of C30G16S16-1 is 14% lower than that of C20G16S16-1, and the *μ*_1_ of C40G16S16-1 is 20% lower than that of C20G16S16-1. The *μ*_2_ of C30G16S16-1 is 26% lower than that of C20G16S16-1, and the *μ*_2_ of C40G16S16-1 is 35% lower than that of C20G16S16-1. As indicated in Table 7, the trend of decreasing ductility with increasing concrete strength is also evident in other axially compressed columns.

## 4. Conclusions

Given that the brittleness of GFRP reinforcement reduces the ductility of the concrete members, this paper employs the hybrid reinforcement of GFRP and SS bars to improve the ductility of axially compressed SWSSC columns. A total of 21 SWSSC columns were subjected to axial compression tests, including 15 hybrid GFRP-SS-reinforced SWSSC (GFRP-SS-SWSSC) columns, 3 GFRP-reinforced SWSSC (GFRP-SWSSC) columns, and 3 SS-reinforced SWSSC (SS-SWSSC) columns. The results were analyzed in terms of failure mode, load–axial displacement curve, load-bearing capacity, and ductility, leading to the following conclusions:

1. The GFRP-SWSSC columns suffered evident brittle failures, while the failures of the GFRP-SS-SWSSC and SS-SWSSC columns showed ductile characteristics. This indicates that hybrid reinforcement improves ductility;
2. The load–axial displacement curves of all the columns showed similar behavior in the ascending part, with the displacement corresponding to the peak load ranging from 0.78 to 0.95 mm, while the descending parts of the columns varied under different reinforcement types. The load for GFRP-SWSSC columns dropped to approximately 50% of the peak load within a few seconds, whereas GFRP-SS-SWSSC and SS-SWSSC columns showed a gradual decline in load. This confirms the beneficial influence of hybrid reinforcement on enhancing ductility.
3. The bearing capacity calculation equation with good accuracy for GFRP-SS-SWSSC axially compressed circular columns was proposed. Additionally, a compressive design strain of 0.002 for GFRP bars was suggested;
4. By analyzing the energy ductility indexes of all the columns, note that the hybrid reinforcement of GFRP and SS bars improves the ductility of the GFRP-SWSSC columns, and the ductility indexes of the GFRP-SS-SWSSC columns are close to those of the SS-SWSSC columns when replacing half of the GFRP bars with SS bars.

## Figures and Tables

**Table 1:** The properties of GFRP bars.

Diameter (mm)	*f*_fu_ (MPa)	COV (%)	*E*_f_ (GPa)	COV (%)
12	914 ± 10.20	1.12	50.3 ± 0.80	1.59
16	889 ± 8.83	0.99	46.8 ± 0.94	2.01
20	870 ± 13.14	1.51	44.2 ± 1.34	3.03

**Table 2:** Primary composition of seawater (g/L).

Cl^−^	Na^+^	SO_4_^2−^	Mg^2+^	Ca^2+^	K^+^
18.61	9.88	2.58	1.17	0.37	0.36

**Table 3:** Mixture proportion of SWSSC (kg/m^3^).

SWSSC Strength Grade	Water	Sand	Stones	Cement
C20	196	775	1161	272
C30	196	697	1185	326
C40	196	634	1175	399

**Table 4:** Mechanical properties of SWSSC.

SWSSC Strength	*f*_cu_ (MPa)	COV (%)	*f*_c_ (MPa)	COV (%)	*E*_c_ (GPa)	COV (%)	*ν*	COV (%)
C20	27.21 ± 0.90	3.31	20.74 ± 1.38	6.65	33.60 ± 1.13	3.36	0.22 ± 0.02	9.09
C30	36.13 ± 0.92	2.55	27.36 ± 1.17	4.28	34.57 ± 1.24	3.59	0.22 ± 0.01	4.54
C40	48.64 ± 1.33	2.73	36.90 ± 1.46	3.96	35.80 ± 2.12	5.92	0.23 ± 0.01	4.35

**Table 5:** The reinforcement details of all the columns.

Column Identifier	Numbers of GFRP and SS Bars	*ρ*_SS_ + *ρ*_GFRP_ (%)	*ρ*_SS_/(*ρ*_SS_ + *ρ*_GFRP_)
C20G16	6 G16	0 + 3.84	/
C20S16	6 S16	3.84 + 0	/
C20G16S16-1	4 G16 + 2 S16	1.28 + 2.56	0.33
C20G16S16-2	3 G16 + 3 S16	1.92 + 1.92	0.50
C20G12G16S16-1	2 G12 + 2 G16 + 2 S16	1.28 + 2.00	0.43
C20G12G16S16-2	3 G12 + 1 G16 + 2 S16	1.28 + 1.72	0.64
C20G16G20S16	2 G16 + 2 G20 + 2 S16	1.28 + 3.28	0.39
C30G16	6 G16	0 + 3.84	/
C30S16	6 S16	3.84 + 0	/
C30G16S16-1	4 G16 + 2 S16	1.28 + 2.56	0.33
C30G16S16-2	3 G16 + 3 S16	1.92 + 1.92	0.50
C30G16S16-3	5 G16 + 1 S16	0.64 + 3.20	0.20
C30G12G16S16-1	2 G12 + 2 G16 + 2 S16	1.28 + 2.00	0.43
C30G12G16S16-2	3 G12 + 1 G16 + 2 S16	1.28 + 1.72	0.64
C40G16	6 G16	0 + 3.84	/
C40S16	6 S16	3.84 + 0	/
C40G16S16-1	4 G16 + 2 S16	1.28 + 2.56	0.33
C40G16S16-2	3 G16 + 3 S16	1.92 + 1.92	0.50
C40G12G16S16-1	2 G12 + 2 G16 + 2 S16	1.28 + 2.00	0.43
C40G12G16S16-2	3 G12 + 1 G16 + 2 S16	1.28 + 1.72	0.64
C40G16G20S16	2 G16 + 2 G20 + 2 S16	1.28 + 3.28	0.39

**Table 6:** The test results of all the columns.

Column Identifier	*P*_max,exp_ (kN)	Δ*u* (mm)	*ε*_cp_ (με)	*ε*_fp_ (με)	*ε*_sp_ (με)	*P*_bar_ (kN)	*P*_bar_/ *P*_max,exp_	*P*_max,pre_ (kN)	*P*_max,pre_/ *P*_max,exp_
C20G16	652.3	0.91	1999	2140	/	120.84	0.19	645.1	0.96
C20S16	798.3	0.81	2026	/	2001	231.67	0.29	766.3	0.96
C20G16S16-1	725.6	0.85	2056	2301	1958	163.85	0.23	684.0	0.94
C20G16S16-2	727.2	0.82	1879	2100	1808	175.13	0.24	700.4	0.96
C20G12G16S16-1	718.5	0.84	2001	G12:2100 G16:2141	1852	141.42	0.20	672.4	0.94
C20G12G16S16-2	712.9	0.90	1983	G12:2004 G16:2060	1917	130.81	0.18	667.3	0.94
C20G16G20S16	766.8	0.82	1810	G16:2024 G20:1895	1717	167.95	0.22	699.9	0.90
C30G16	763.4	0.95	1755	1897	/	107.12	0.14	779.5	1.02
C30S16	887.8	0.79	1945	/	1940	231.67	0.26	900.6	1.01
C30G16S16-1	806.2	0.78	1829	2087	1787	155.79	0.19	804.3	0.95
C30G16S16-2	828.4	0.81	1981	2291	2098	180.52	0.22	848.2	1.02
C30G16S16-3	802.6	0.85	1920	2058	1877	135.46	0.17	797.7	0.99
C30G12G16S16-1	797.3	0.87	1841	G12:2005 G16:1928	1974	136.33	0.17	807.6	1.01
C30G12G16S16-2	779.5	0.78	1846	G12:1979 G16:2133	1939	131.07	0.17	803.0	1.03
C40G16	946.7	0.79	2050	2178	/	122.99	0.13	1012.7	1.07
C40S16	1179.6	0.81	1987	/	1972	231.67	0.20	1133.9	0.96
C40G16S16-1	1039.4	0.85	2231	2359	2149	166.03	0.16	1058.7	1.02
C40G16S16-2	1095.4	0.90	1970	2010	1981	172.58	0.16	1073.6	0.98
C40G12G16S16-1	1015.5	0.81	2085	G12:2281 G16:2217	2040	144.91	0.14	1042.3	1.03
C40G12G16S16-2	974.1	0.84	1980	G12:2187 G16:2117	1980	134.47	0.14	1038.5	1.07
C40G16G20S16	1058.6	0.80	1867	G16:2078 G20:1987	1972	171.53	0.16	1053.1	0.99
Average	/	/	1954	2088	1942	/	/	/	0.99

Note: *P*_max,exp_ is the experimental peak load; Δ*u* denotes the axial displacements at the peak load; *ε*_fp_, *ε*_sp_, and *ε*_cp_ are the strains of the GFRP and SS bars and concrete at the peak load, respectively; *P*_bar_ is the bearing capacities provided by the longitudinal bars; and *P*_max,pre_ and is the predicted bearing capacity.

**Table 7:** Calculation parameters and the calculated ductility indexes.

Column Identifier	Δ_y_ (mm)	Δ_75_ (mm)	Δ_85_ (mm)	*E*_y_ (J)	*E*_u_ (J)	*μ*_1_	*μ*_2_
C20G16	0.37	0.44	1.43	143.4	396.1	2.76	3.86
C20S16	0.36	0.58	2.46	210.7	759.5	3.61	6.83
C20G16S16-1	0.33	0.53	2.02	186.7	624.2	3.34	6.12
C20G16S16-2	0.31	0.48	1.98	180.2	624.2	3.47	6.30
C20G12G16S16-1	0.31	0.46	1.71	182.2	585.1	3.21	5.52
C20G12G16S16-2	0.38	0.51	1.93	166.5	521.7	3.13	5.12
C20G16G20S16	0.32	0.48	2.01	182.0	648.4	3.56	6.36
C30G16	0.39	0.48	1.40	183.1	448.0	2.45	3.59
C30S16	0.35	0.55	2.13	243.9	802.7	3.29	6.01
C30G16S16-1	0.35	0.44	1.58	193.7	555.6	2.87	4.51
C30G16S16-2	0.31	0.51	1.60	185.7	568.6	3.06	5.23
C30G16S16-3	0.38	0.50	1.46	208.6	525.6	2.52	3.84
C30G12G16S16-1	0.33	0.45	1.41	193.1	538.4	2.79	4.27
C30G12G16S16-2	0.44	0.48	1.59	183.4	490.6	2.68	3.61
C40G16	0.36	0.44	1.23	208.1	493.2	2.37	3.42
C40S16	0.38	0.58	1.98	341.8	991.8	2.90	5.21
C40G16S16-1	0.46	0.56	1.83	321.6	860.3	2.68	3.98
C40G16S16-2	0.45	0.58	1.86	285.8	789.2	2.76	4.13
C40G12G16S16-1	0.45	0.53	1.54	255.7	637.3	2.49	3.42
C40G12G16S16-2	0.43	0.49	1.39	256.6	624.8	2.43	3.23
C40G16G20S16	0.42	0.59	1.96	306.4	865.2	2.82	4.67

## Data Availability

Data are contained within the article.
